# Predatory *Myxococcus fulvus* strain KS01 confers biocontrol and growth promotion in cotton through multifaceted antifungal mechanisms

**DOI:** 10.3389/fmicb.2026.1812103

**Published:** 2026-06-17

**Authors:** Jian Han, Wen Pan, Ming Luo, Benzhong Fu, Liqun Zhang

**Affiliations:** 1 Department of Plant Pathology, College of Agronomy, Xinjiang Agricultural University, Urumqi, China; 2 Key Laboratory of Prevention and Control of Invasive Alien Species in Agriculture & Forestry of the North-Western Desert Oasis (Co-Construction by Ministry and Province), Ministry of Agriculture and Rural Affairs, Urumqi, China; 3 Key Laboratory of the Pest Monitoring and Safety Control of Crops and Forests of the Xinjiang Uygur Autonomous Region, Urumqi, China; 4 College of Plant Protection, China Agricultural University, Beijing, China

**Keywords:** antifungal activity, biological control, cotton verticillium wilt, myxobacteria, *Myxococcus fulvus*, predation, *Verticillium dahliae*

## Abstract

Cotton Verticillium wilt, caused by *Verticillium dahliae*, is one of the most destructive soil-borne fungal diseases threatening cotton production worldwide, necessitating the development of effective and sustainable control strategies. Myxobacteria are higher-order prokaryotes characterized by multicellular social behavior and predatory activity against diverse microorganisms, conferring significant potential for plant disease management. Based on a previously established myxobacterial mini-resource library, a strain designated KS01 with broad-spectrum antifungal activity was screened and characterized. Morphological, conservative genes (16S rRNA and *gyrB*) and genomic core genes phylogenetic tree identified the strain as *Myxococcus fulvus* KS01. *In vitro* assays demonstrated that *M. fulvus* KS01 lysed hyphae and spores of *V. dahliae* and significantly inhibited spore germination. Both sterile fermentation filtrates and volatile organic compounds (VOCs) exhibited antifungal activity. While crude secondary metabolite extracts (via macroporous resin adsorption) and crude lipopeptide extracts (via acid precipitation) exhibited no inhibitory activity, the ammonium sulfate–precipitated extracellular crude protein fraction displayed pronounced antifungal activity. This fraction disrupted fungal cell wall and membrane integrity, induced intracellular reactive oxygen species (ROS) accumulation, lysed spores, and suppressed germ tube elongation. Substrate spectrum analysis further revealed that KS01 produces peptidases, lipases, and glycoside hydrolases, which are likely key biocontrol determinants. Greenhouse pot experiments showed that a solid formulation of *M. fulvus* KS01, prepared using white star flower chafer (*Protaetia brevitarsis*) frass as the fermentation substrate, achieved a control efficacy of 63.01% against cotton Verticillium wilt and significantly promoted cotton seedling growth. Collectively, *M. fulvus* KS01 represents a promising predatory biocontrol agent with substantial potential for the sustainable management of cotton Verticillium wilt.

## 1 Introduction

Verticillium wilt of cotton, caused by *Verticillium dahliae*, is one of the most destructive soil-borne vascular diseases in major cotton-producing regions worldwide, causing substantial yield losses and deterioration of fiber quality (Zhang et al., 2026). Current control strategies face two major challenges: chemical control is compromised by the emergence of pathogen resistance, disruption of soil microbial communities, and environmental contamination; and breeding for resistance is time-consuming and often undermined by rapid pathogen evolution (Wang G. et al., 2020). Against this backdrop, environmentally benign biological control approaches have attracted increasing interest as sustainable alternatives.

Existing biological control efforts have focused primarily on a limited set of microbial taxa. Fungal biocontrol agents are dominated by genera such as *Trichoderma* and *Chaetomium* (Zhang et al., 2021; Kong et al., 2022), while bacterial agents are typically represented by *Bacillus* and *Streptomyces* species (Lyu et al., 2019; Zeng et al., 2019). Reliance on these “well-studied” strains has constrained the taxonomic and functional diversity of available biocontrol repositories, motivating the search for novel antagonists with distinct modes of action and improved field performance.

Myxobacteria are social, predatory bacteria with broad environmental distributions, including organic-rich soils, decaying wood, animal dung, and marine habitats (Wrótniak-Drzewiecka et al., 2016). They employ coordinated gliding motility and group predation to lyse and consume other microorganisms, and when nutrients become limiting they form multicellular fruiting bodies that produce resistant myxospores capable of surviving adverse conditions (Munoz-Dorado et al., 2016). Myxobacteria are also prolific producers of structurally diverse secondary metabolites and extracellular hydrolytic enzymes—a metabolic repertoire second only to actinomycetes and *Bacillus* among prokaryotes—making them a promising reservoir of natural products for agriculture and medicine (Zhang et al., 2024). Ecologically, myxobacteria occupy a top-down position in the soil microbial food web; their predatory activity can directly affect soilborne pathogen populations and thereby influence soil microecology and plant health (Dai et al., 2024). These attributes point to substantial, yet underexplored, potential for myxobacteria in plant disease biocontrol.

Empirical evidence from greenhouse and field studies supports the biocontrol potential of myxobacteria against diverse plant pathogens. For instance, six isolates of *Myxococcus* exhibited lytic and predatory activity against eight soilborne fungal pathogens and could protect lettuce from *Sclerotinia minor* (Bull et al., 2002). Decade ago, 30 myxobacterial strains isolated from forest soils demonstrated capacity of protecting pine seedlings from *Rhizoctonia solani*; among several strains showed good colonization ability in potting soil (Dahm et al., 2015). More recent work includes the screening of *M. xanthus* R31, which displayed strong antagonism against *Ralstonia solanacearum* and achieved an 81.9% control efficiency of bacterial wilt on tomato in pot experiments (Dong et al., 2021). Field trials have also produced encouraging results: *Sorangium cellulosum* KYC 3262 provided stable control of pepper anthracnose over three consecutive years, with efficacy comparable to chemical fungicides (Yun, 2014), and *Corallococcus* sp. EGB outperformed chemical treatments in 2 years of cucumber and banana wilt field trials, substantially increasing crop yield (Ye et al., 2020a,b).

In this study, we isolated a myxobacterial strain, KS01, from a saline–alkaline cotton field in Yuli County, Xinjiang Uygur Autonomous Region, China, and evaluated its antagonistic and predatory activity against *V. dahliae in vitro*. KS01 was taxonomically characterized and its biocontrol potential was assessed in greenhouse pot experiments. We further conducted preliminary investigations into the mechanisms underlying KS01’s antagonism, including the effects of cell-free fermentation broth, volatile compounds, extracellular metabolites, and hydrolytic enzymes on *V. dahliae* hyphae and spores. The results provide a foundation for exploiting myxobacteria as novel biocontrol agents against cotton Verticillium wilt and for future development of green, targeted crop protection strategies.

## 2 Materials and methods

### 2.1 Strains, media, and plant material

Six phytopathogenic fungal strains used in this study were obtained and are listed with GenBank accession numbers where available: *V. dahliae* (Vd, KC282468) and *Fusarium oxysporum* f. sp. *vasinfectum* (Fov, KR071660) were kindly provided by Prof. Aixing Gu (Xinjiang Agricultural University); *Alternaria tenuissima* (At, OM884060) and *Fusarium culmorum* (Fc, OP315277) were kindly provided by Dr. Lili Wang (Xinjiang Agricultural University); *Fusarium verticillioides* (Fv, OR105502) and *R. solani* (Rs, HF912170) were kindly provided by Prof. Qingyuan Guo (Xinjiang Agricultural University). All strains are maintained in the Agricultural Microbiology and Biotechnology Laboratory, College of Agriculture, Xinjiang Agricultural University.

Activation and antagonism screening of myxobacteria were performed on VY/2 medium. Liquid cultures of myxobacteria were grown in LBS medium. Pathogenic fungi were cultured and sporulated on Potato Dextrose Agar (PDA) and Czapek–Dox medium. Preparation of VY/2, LBS, PDA and Czapek–Dox media followed the recipes described by Han et al. (2025).

The cotton cultivar used for biocontrol efficacy assays was *Gossypium hirsutum* cv. “Xinluzao 18” (Verticillium-wilt resistant), supplied by Dr. Dawei Zhang, Institute of Cash Crops, Xinjiang Academy of Agricultural Sciences.

### 2.2 Isolation and morphological observation of myxobacteria

Soil samples were collected from a saline–alkaline cotton field in Yuli County, Xinjiang Uygur Autonomous Region, China (86°03′48″E, 41°48′35″N). Isolation of myxobacteria followed the soil baiting and transfer method described by Zhang et al. (2003). In brief, fruiting bodies induced from sterile rabbit dung pellets; subsequently transfer the fruiting-body to new VY/2 plates, incubated at 30 °C for 5 d to obtain colony. When spreading colonies or fruiting bodies were observed (typically after ~5 days), colony margins were picked and transferred to fresh VY/2 plates; this subculture procedure was repeated until axenic cultures were obtained.

Pure cultures of the isolate designated KS01 were incubated on VY/2 agar for 5 days for colony morphology documentation and imaging. Fruiting bodies were examined using a stereomicroscope (SM7, Motic China Group Co., Ltd.). Vegetative cells and myxospores were examined by Gram staining and bright-field microscopy (Ni-U, Nikon). For scanning electron microscopy (SEM), cells and fruiting bodies were fixed in 2.5% glutaraldehyde, processed according to standard protocols, and imaged with a Zeiss SUPRA55VP SEM at the Laboratory Center, Xinjiang Institute of Ecology and Geography, Chinese Academy of Sciences.

### 2.3 16S rRNA and *gyrB* gene analysis

Total genomic DNA was extracted from KS01 using the TIANamp Bacteria DNA Kit (TIANGEN Biotech (Beijing) Co., Ltd., China) according to the manufacturer’s instructions. The nearly full-length 16S rRNA gene was amplified with universal primers 27F (5′-AGAGTTTGATCCTGGCTCAG-3′) and 1492R (5′-TACGGCTACCTTGTTACGACTT-3′) (Kumar et al., 2017). The partial *gyrB* gene (DNA gyrase subunit B) was amplified with primers *gyrB*F (5′-GCGGAAGCGGCCNGSNATGTA-3′) and *gyrB*R (5′-CCGTCCACGTCGGCRTCNGYCAT-3′) (Santos and Ochman, 2004). PCR amplicons were purified and sequenced by Sanger sequencing (Sangon Biotech (Shanghai) Co., Ltd., China).

Resulting sequences were compared to publicly available sequences in the NCBI database using BLAST. Multiple sequence alignments and phylogenetic analyses were performed in MEGA v11.0. Phylogenetic trees for 16S rRNA and *gyrB* were constructed using the Neighbor-Joining method in MEGA 11.0.

### 2.4 Whole-genome sequencing, assembly and genome-scale analyses

#### 2.4.1 DNA extraction, sequencing and assembly

The genomic DNA of strain KS01 was extracted using a commercial DNA kit (TIANGEN Biotech (Beijing) Co., Ltd., China). A hybrid whole-genome shotgun strategy was used: long-read sequencing (Oxford Nanopore Technologies, ONT) for scaffolding and short-read Illumina sequencing for base-level correction. Raw reads were quality-filtered (Illumina reads with *fastp*; ONT reads with platform-appropriate filters). *De novo* and hybrid assemblies were produced using hybridSPAdes (Antipov et al., 2016). Assemblies were polished with *Pilon* using Illumina reads (Chen et al., 2021). Genome completeness/contamination was assessed with *CheckM* (Parks et al., 2015).

#### 2.4.2 Structural and functional annotation

Structural annotation (CDSs, tRNAs, rRNAs and other ncRNAs) was performed with automated pipelines (NCBI PGAP or Prokka); tRNAs were predicted with *tRNAscan-SE*, rRNAs with *Barrnap*, and other ncRNAs by comparison to the *Rfam* database. Functional annotation included assignment to COG and KEGG pathways. Secondary-metabolite biosynthetic gene clusters were detected using antiSMASH (v7.0). Carbohydrate-active enzymes (CAZymes) were predicted by dbCAN3/hmmscan against the CAZy database to identify families relevant to fungal cell-wall degradation (e.g., GH18/GH19 chitinases, β-1,3-glucanases). Proteases and other hydrolytic enzymes were annotated by homology searches and curated against relevant databases.

#### 2.4.3 Comparative genomics and gene mining for predation-related systems

Average nucleotide identity (ANI) comparisons were performed with fastANI to identify nearest relatives. Core-gene phylogenomics was inferred using the UBCG core gene set. Secreted proteins were predicted with SignalP 6.0 (signal peptides) and TMHMM 2.0 (transmembrane helices); proteins with signal peptides and no transmembrane regions outside the signal peptide were classed as likely secreted. Secretion systems were identified with MacSyFinder and manual inspection.

### 2.5 Antifungal activity of strain KS01

Strain KS01 was activated on VY/2 agar for 5 days. A confluent portion of colony was scraped and inoculated into 50 mL LBS broth, incubated at 30 °C with shaking at 180 rpm for 48 h. This pre-culture was used to inoculate 200 mL LBS broth at 3% (v/v) and incubated at 30 °C, 180 rpm for 72 h to obtain the KS01 fermentation broth. Cells were pelleted by centrifugation at 12,000 rpm for 1 min, washed three times with sterile water, dispersed thoroughly, and resuspended in sterile water to an OD₆₀₀ of 2.0.

Antagonistic assays were performed on VY/2 agar. Five-mm agar plugs (*d* = 5 mm) of each phytopathogenic fungus (*V. dahliae*, *A. tenuissima*, *F. oxysporum* f. sp. *vasinfectum*, *F. verticillioides*, *R. solani*, *F. culmorum*) were placed at the center of VY/2 plates. KS01 cell suspension (20 μL; OD₆₀₀ = 2.0) was spotted at four positions (approximately 2 cm from the edge of the fungal plug: above, below, left and right). Plates were incubated at 28 °C for 7 days, photographed and scored. Plates without KS01 served as controls. Each treatment comprised three replicate plates, and the experiment was repeated three times.

### 2.6 Lytic activity of KS01 against *V. dahliae* hyphae

#### 2.6.1 Hyphal assays on solid media

Sterile dialysis tubing (3.5 kDa MWCO) was laid flat on VY/2 agar. A 5 mm agar plug of *V. dahliae* was placed at the center and incubated at 28 °C until the colony reached ~1.0 cm in diameter. KS01 suspension (20 μL; OD₆₀₀ = 2.0) was then applied in a linear series approximately 1 cm from the hyphal margin. Plates were incubated at 28 °C for 5 days. Dialysis tubing was then removed and myxobacterial and fungal material sampled from distinct regions for microscopy.

#### 2.6.2 Hyphal assays in liquid co-culture

*V. dahliae* agar plugs were transferred to potato dextrose broth (PDB) and incubated statically at 26 °C for 5 days. Mycelia were collected by centrifugation at 10,000 rpm for 15 min. For co-culture, 0.1 g (wet weight) of *V. dahliae* mycelia was mixed with 1 mL KS01 suspension (OD₆₀₀ = 2.0) in a 25 mL co-culture system consisting of 15 mL TPM buffer (10 mM Tris–HCl, 1 mM KH_2_PO_4_, 8 mM MgSO_4_·7H_2_O), 5 mL LBS broth and 5 mL PDB. Sterile water replaced KS01 suspension in control treatments. Co-cultures were incubated at 28 °C with shaking at 180 rpm for 48 h.

Samples from both solid and liquid assays were fixed in 2.5% glutaraldehyde and observed by scanning electron microscopy (Zeiss SUPRA55VP) to document hyphal damage and myxobacterial interactions. Each treatment included three biological replicates, and each biological replicate was observed at three randomly selected fields of view.

### 2.7 Greenhouse evaluation of KS01 solid formulation against cotton verticillium wilt

#### 2.7.1 Preparation of KS01 solid formulation and *V. dahliae* inoculum

KS01 fermentation broth (prepared as in Section 2.5) was adjusted to OD₆₀₀ = 2.0 and used as seed inoculum for solid fermentation. Sterile solid substrate composed of white star flower chafer frass (provided by Jinkun Insect Biotechnology Co., Ltd. Xinjiang) mixed with wheat straw at a 3:1 (w/w) ratio to serve as the solid-state fermentation substrate, which was sterilized at 121 °C (0.1 MPa) for 30 min. The substrate was inoculated with the myxobacterial fermentation broth at 50 mL·kg^−1^, mix thoroughly, and adjusted to a final moisture of 60%. Following incubation at 30 °C for 6 days, the final myxospore concentration in the solid inoculant reached 10^5^ CFU/g.

*V. dahliae* was cultured on PDA at 28 °C for 7 days. Four 5 mm plugs were inoculated into 200 mL Czapek broth and shaken at 200 rpm, 28 °C for 6 days. The culture was filtered through four layers of sterile gauze to collect spores. Spore suspensions were adjusted to 1.0 × 10^8^ spores·mL^−1^ and stored on ice until use.

#### 2.7.2 Greenhouse pot experiment

Cotton seedlings (cv. Xinluzao 18) were prepared as described by Wei et al. (2019). Six treatments were arranged as follows: (1) SFK + Vd—KS01 solid formulation applied at sowing (1.0 g placed beneath each seed and covered with ~1 cm soil) followed by *V. dahliae* inoculation; (2) SSFS + Vd—sterile substrate control applied as in (1) plus *V. dahliae* inoculation; (3) Vd—*V. dahliae* only; (4) SFK—KS01 solid formulation only; (5) SSFS—sterile substrate only; (6) Mock—healthy control with no treatment.

At sowing, treatments (1) and (2) received 1.0 g substrate per seed as described. At the two-leaf stage, lateral roots were wounded with a sterile needle and 5 mL of *V. dahliae* spore suspension (1.0 × 10^8^ spores·mL^−1^) was applied to the rhizosphere for inoculated treatments (1–3). Ten seedlings were planted per pot; three pots constituted one treatment (i.e., three replicate pots per treatment). Pots were arranged randomly in the greenhouse; the experiment was conducted once with three replicates per treatment. The potting assay was repeated three times independently. Greenhouse conditions: 25–32 °C, relative humidity >60%. Standard watering and fertilization regimes were applied.

#### 2.7.3 Agronomic measurements and disease assessment

Fifty days after emergence, plant height, stem diameter, primary root length, shoot fresh weight, root fresh weight, shoot dry weight and root dry weight were measured for Mock, SFK and SSFS treatments. Thirty days after *V. dahliae* inoculation, vascular bundle discoloration and disease incidence were recorded. Disease severity was scored using the five-grade scale for cotton Verticillium wilt (Xue et al., 2013). Disease index and control efficacy were calculated as follows:


$$\begin{array}{l} \text{Disease index}=\\ \frac{\sum (\text{number of plants}\;\mathrm{at}\;\text{each grade}\times \text{grade value})}{\text{total number of plants}\times \text{highest grade}}\times 100 \end{array}$$

$$\begin{array}{l} \text{Control efficacy}(\%)=\\ \frac{\text{Disease}\;{\text{index}}_{\text{control}}-\text{Disease}\;{\text{index}}_{\text{treatment}}}{\text{Disease}\;{\text{index}}_{\text{control}}}\times 100 \end{array}$$

All data are expressed as mean ± SD. Statistical analyses were performed using SPSS.

### 2.8 Antifungal activity of cell-free filtrate and VOCs

#### 2.8.1 Preparation of cell-free filtrate

KS01 fermentation broth was prepared as in Section 2.5. Broth was centrifuged at 12,000 rpm for 15 min at 4 °C and the supernatant filtered through a 0.22 μm MCE membrane (Tianjin Linghang). The sterile filtrate was incorporated into VY/2 agar at 40% (v/v); sterile LBS served as the control.

#### 2.8.2 Plate assay

A 5 mm plug of *V. dahliae* was placed at the center of each plate; colony diameter was measured after incubation at 28 °C for 7 days. Three replicate plates per treatment and the all experiments were repeated three times.

#### 2.8.3 Spore germination and lysis assay

Mix 1 mL sterile filtrate with 1 mL *V. dahliae* spore suspension (1.0 × 10^8^ spores·mL^−1^) and incubate at 28 °C, 180 rpm. Controls received sterile LBS. At 24 and 48 h, assess spore germination with a hemocytometer and calculate spore lysis and germination inhibition rates (Xue et al., 2013). Three technical replicates per treatment and the all experiments were repeated three times.


$$\text{Spore lysis rate}(\%)=\frac{N_{\text{control}}-N_{\text{treatment}}}{N_{\text{control}}}\times 100$$

$$\text{Spore germination inhibition rate}(\%)=\frac{G_{\text{control}}-G_{\text{treatment}}}{G_{\text{control}}}\times 100$$

Where N = total spores counted and G = percentage germinated (germinated spores per 100 spores × 100).

#### 2.8.4 VOCs assay

Following Wu et al. (2015) protocol, 100 μL KS01 suspension (OD₆₀₀ = 1.0) was spread on VY/2 agar and paired (face-to-face) with a PDA plate inoculated centrally with a 5 mm *V. dahliae* plug. Paired plates were sealed with film and incubated at 28 °C for 10 days. A VY/2 agar plate without bacterial inoculation was paired with a *V. dahliae* plate as the negative control. Colony diameter was recorded. Three replicates for each treatment and the experiment was repeated three times.

### 2.9 Antifungal activity of secreted metabolites

#### 2.9.1 Metabolite preparation

*Crude secondary metabolites*: XAD-16 resin (Amberlite XAD-16N, 20–60 mesh) was added to sterile KS01 fermentation broth to adsorb metabolites. Resin was eluted with methanol; eluate was rotary-evaporated to dryness (35 °C, 40 rpm, reduced pressure) and reconstituted in PBS to 100 mg·mL^−1^. Filtered through 0.22 μm membrane prior to use.

*Crude lipopeptides*: Adjust filtrate pH to 2.0 with 6 M HCl; precipitate at 4 °C for 12 h, centrifuge at 12,000 rpm, 4 °C for 30 min. Dissolve precipitate in methanol, evaporate and reconstitute in PBS to 500 mg·mL^−1^.

*Crude protein fraction*: The fermentation supernatant (1 L) was brought to ~100% saturation with ammonium sulfate and incubated at 4 °C for 12 h to allow protein precipitation. The precipitate was collected by centrifugation at 12,000 rpm for 30 min, resuspended in an appropriate volume of 10 mM PBS (pH 7.4), and subjected to dialysis using a 3.5 kDa molecular weight cutoff membrane against 10 mM PBS at 4 °C for 16 h. The dialyzed protein solution was subsequently filtered through a 0.22 μm membrane, and protein concentration was determined using a BCA assay (BCAP-1-W, Suzhou KeMing).

#### 2.9.2 Bioassays

*Oxford-cup plate assay*: A 5 mm *V. dahliae* plug was placed at the center of PDA. Two Oxford cups were symmetrically positioned 2 cm from the plug and loaded with 100 μL of each test sample (secondary metabolite crude extract, lipopeptide extract, crude protein). PBS served as blank control. Plates were incubated at 28 °C for 7 days and colony diameters measured. Three replicates for each treatment and the experiment was repeated three times.

*Liquid exposure microscopy*: For each treatment, 1 mL test extract (as above) was mixed with 1 mL *V. dahliae* spore suspension (1.0 × 10^8^ spores·mL^−1^), incubated at 30 °C, 180 rpm for 24 h; PBS was control. Hyphae and spores were examined by light microscopy (Ni-U, Nikon).

### 2.10 Effects of crude extracellular protein on cell wall, membrane and ROS

*Co-culture*: Combine 1 mL crude extracellular protein solution (3.1 mg·mL^−1^) with 0.1 g fresh *V. dahliae* mycelia in a 25 mL co-culture system (as Section 2.6). PBS (1 mL) served as control. Incubate at 30 °C, 180 rpm; sample hyphae at 6 and 12 h. Each treatment was performed in three biological replicates.

*Cell-wall integrity (calcofluor white)*: Place a small amount of hyphae into 10 μL Calcofluor White M2R solution (1 g·L^−1^) + 10 μL KOH (0.18 mol·L^−1^); incubate 1 min and observe under fluorescence microscope (Jofré et al., 2018). Triplicate samples per time point.

*Membrane integrity (PI)*: Stain hyphae with 10 μL propidium iodide (5 μg·mL^−1^) at room temperature in the dark for 30 min; wash 2-3 × with sterile water and observe by fluorescence microscopy (Ye et al., 2020b). Triplicate samples per time point.

*ROS accumulation (DCFH-DA)*: Suspend hyphae in 90 μL PBS, add 10 μL DCFH-DA (stock 1 mg·mL^−1^), incubate at 28 °C for 30 min, and observe by confocal laser scanning microscopy (AXR CLSM, Nikon). Triplicate samples per time point.

All staining and fluorescence protocols followed manufacturer instructions; imaging performed with identical settings across treatments.

### 2.11 Extracellular enzyme assays and substrate spectrum

Qualitative plate assays. 10 μL of KS01 culture suspension (OD₆₀₀ = 1.0) was spotted onto substrate-specific plates containing colloidal chitin, *β*-glucan, skim milk, starch, carboxymethyl cellulose (CMC), and tributyrin. Plates were incubated at 30 °C for 5 days. Each substrate tested on three plates and the experiment was repeated three times. Zones of clearance or dye changes were recorded.

Quantitative assays. Polysaccharide-degrading activities (chitin, pustulan, CMC, xylan, yeast glucan, β-1,3-glucan) were quantified using the DNS method on plate extracts following Hu et al. (2008) and a DNS Assay Kit (Beijing Solarbio). Lipase activity was assayed with p-nitrophenyl palmitate substrate (Zheng et al., 2011) and quantified against a p-nitrophenol standard curve. Inactivated enzyme (100 °C, 10 min) served as negative control. All assays were performed in triplicate.

### 2.12 Statistical analysis

All data are presented as mean ± standard deviation (SD). Statistical comparisons were performed by one-way ANOVA followed by Duncan’s multiple range test using SPSS v19.0; significance was set at *p* < 0.05. Graphs and additional data visualization were produced in GraphPad Prism 8. Sample sizes and number of replicates are stated for each assay above.

## 3 Results

### 3.1 Characterization and identification of strain KS01

**Figure 1:**
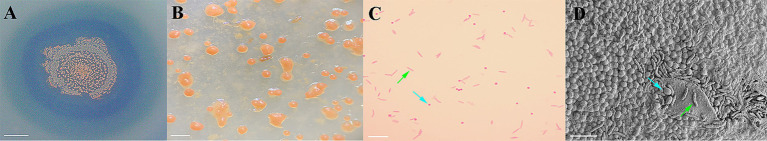
Colony morphology, Gram stain, and scanning electron micrograph (SEM) of strain KS01. **(A)** Colony morphology of strain KS01 on VY/2 agar. Scale bar = 5 mm. **(B)** Fruiting bodies produced by KS01 on VY/2 agar after 5 days of incubation. Scale bar = 0.5 mm. **(C)** Vegetative cell and myxospore morphology of KS01 after Gram staining, observed by light microscopy. Scale bar = 20 μm. **(D)** SEM micrograph showing vegetative cells and myxospores of KS01. Green arrows indicate vegetative cells; blue arrows indicate myxospores. Scale bar = 5 μm.

A myxobacterial isolate, designated KS01, was recovered from saline–alkaline cotton soil using rabbit-dung baiting. On VY/2 agar KS01 formed thin, spreading films (Figure 1A). Fruiting bodies were predominantly spherical, solitary and orange (Figure 1B). Vegetative cells were slender rods and myxospores were spherical, as shown by light microscopy and SEM (Figures 1C,D).

**Figure 2:**
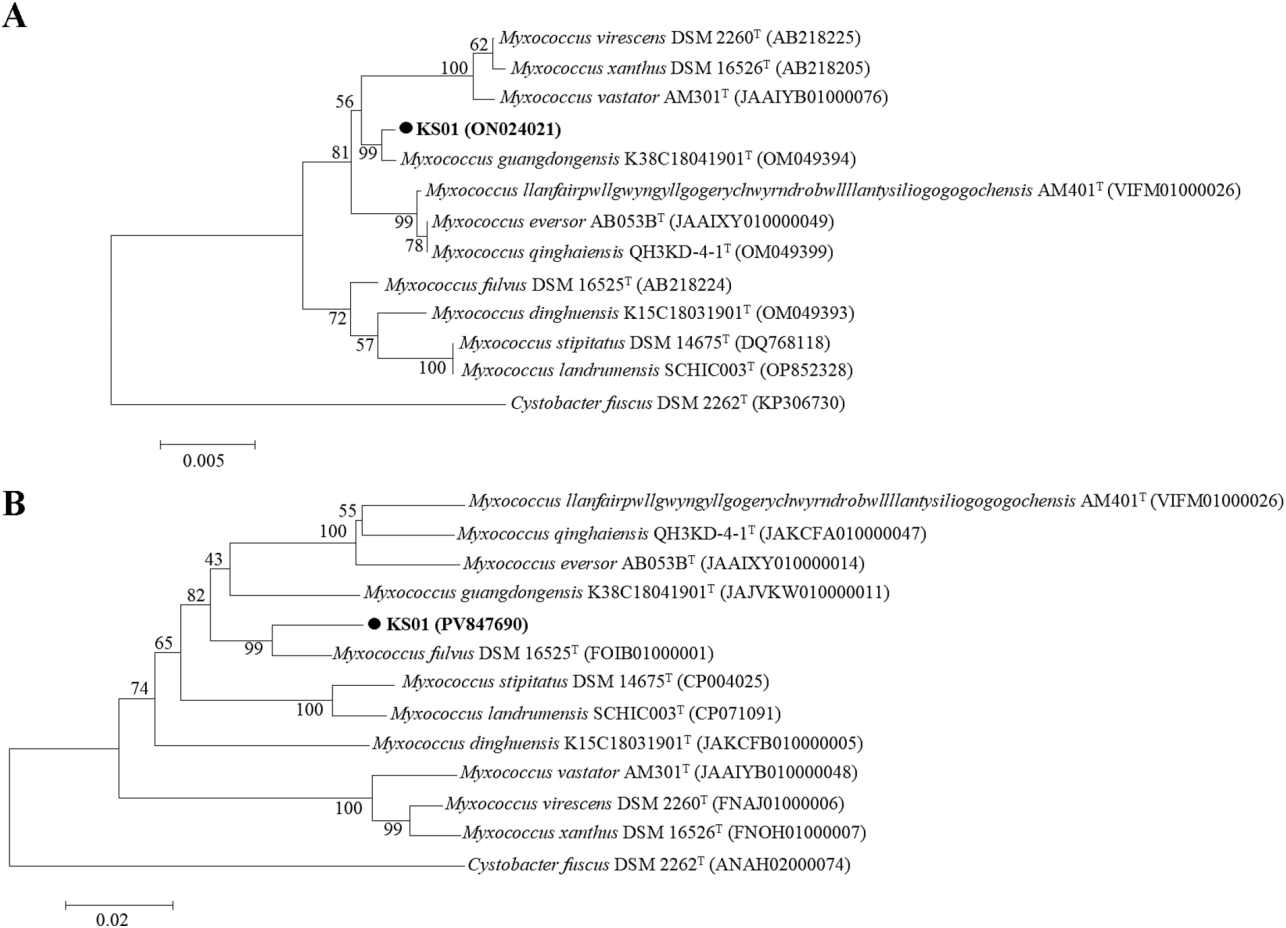
Phylogenetic trees of strain KS01 based on 16S rRNA and *gyrB* sequences. **(A)** Based on 16S rRNA gene sequences. **(B)** Based on *gyrB* gene sequences. Trees were constructed in MEGA v11.0 using the neighbor-joining method; bootstrap = 1,000.

The nearly complete 16S rRNA and partial *gyrB* genes were PCR-amplified and sequenced (GenBank accession numbers: 16S rRNA ON024021; *gyrB* PV847690). Phylogenetic analysis based on 16S rRNA placed KS01 within the *Myxococcus* clade (Figure 2A). A *gyrB*-based tree further clustered KS01 with *M. fulvus* DSM 16525^T^ (FOIB01000040.1) (Figure 2B). Considering morphology and multilocus sequence evidence, KS01 was identified as *M. fulvus*.

### 3.2 Characterization of the KS01 genome

#### 3.2.1 General genomic features

**Figure 3:**
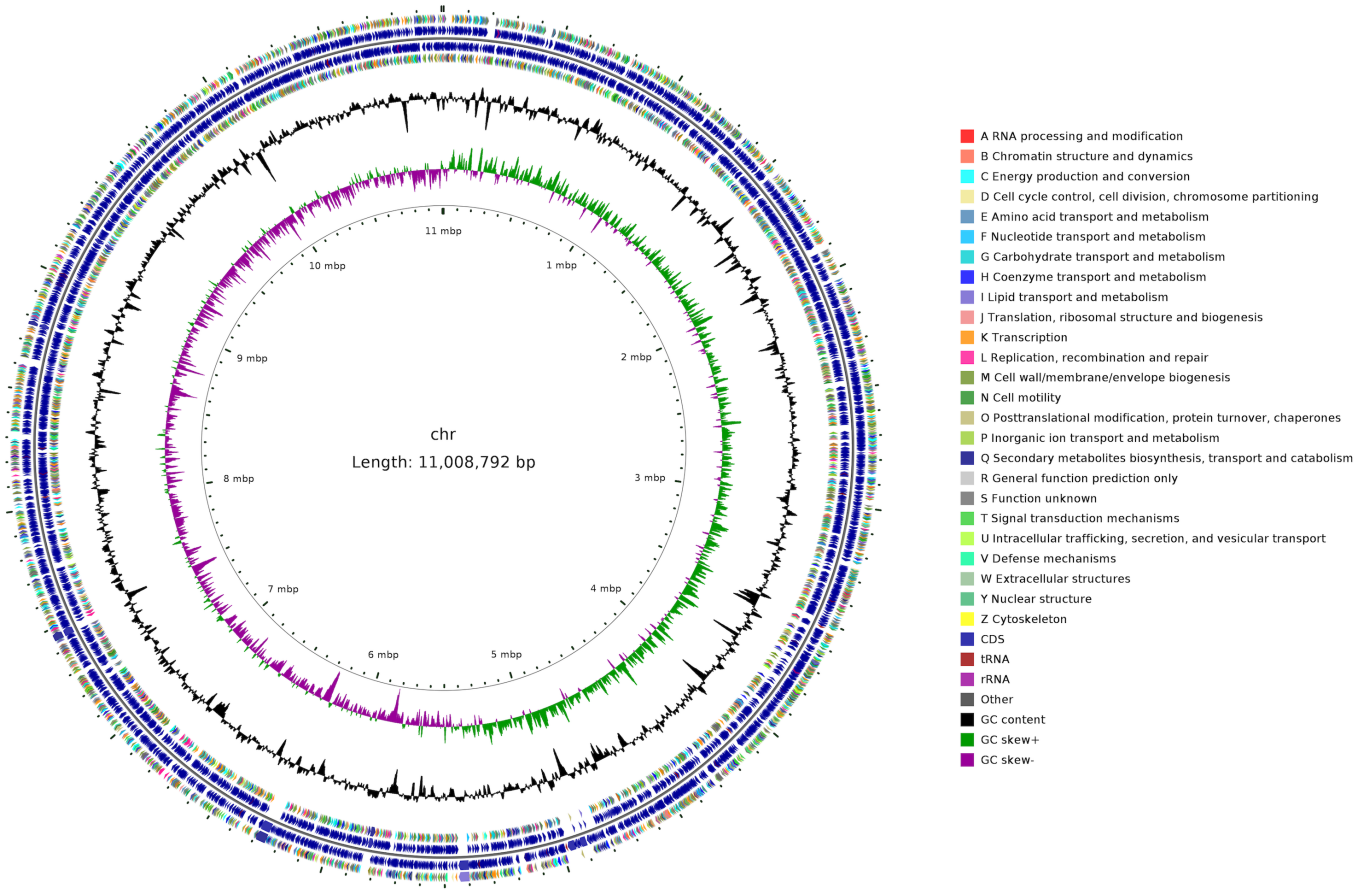
Genome map of KS01 (CGView). From inside to out: (1) scale, (2) GC skew, (3) GC content, (4 & 7) COG category for each CDS, and (5 & 6) positions of CDS, tRNA, and rRNA.

The KS01 genome was sequenced using Illumina NovaSeq (paired-end, 2 × 150 bp) and Nanopore platforms and assembled into a high-quality, complete circular chromosome (Figure 3). The chromosome is 11,008,792 bp in length with a GC content of 69.90% and an estimated completeness of 99.35%. No plasmids were detected.

A total of 8,670 open reading frames (ORFs) were predicted, with a cumulative ORF length of 9,779,532 bp, corresponding to a coding percentage of 88.83%. The ORF density is 0.788 genes kb^−1^ and the mean ORF length is 1,127.97 bp (longest ORF = 45,453 bp). The GC content of coding sequences (70.24%) is slightly higher than that of the whole genome. In addition to 5S, 16S, and 23S rRNA genes, the genome contains 87 tRNA genes and 13 noncoding RNA (ncRNA) genes.

A phylogenetic tree was constructed from 92 single-copy core genes conserved across representative species (Supplementary Figure 1). KS01 clustered with *M. fulvus* (GCF_900111765.1); 91 core genes support this branch. Average nucleotide identity (ANI) analysis against 20 closely related genomes produced an ANI of 97.42% with *M. fulvus* (GCF_900111765.1). Based on these genome-level metrics, KS01 is identified as *M. fulvus*.

#### 3.2.2 Identification of potential antifungal compounds and secretion/predation mechanisms

Protein sequences were scanned against a local CAZy database using HMMER (hmmscan). Six CAZy classes were detected; the most abundant class was glycosyltransferases (GTs) with 92 genes, followed by glycoside hydrolases (GHs, 71), carbohydrate esterases (CEs, 70), carbohydrate-binding modules (CBMs, 52), polysaccharide lyases (PLs, 20), and auxiliary activities (AAs, 16). antiSMASH predicted a terpene biosynthetic gene cluster of 22,265 bp showing 100% similarity to a geosmin terpene cluster. Signal peptide prediction identified three proteins with classical signal peptides and two proteins with lipoprotein signal peptides, their probabilities ranged from 0.9797 to 1.0000 algin with the database. MacSyFinder detected a Type VI secretion system (T6SSi) cluster in the genome. T6SSi (Type VI secretion system subtype i) is consistent with canonical T6SS architectures and is commonly implicated in contact-dependent antagonism, which may contribute to intermicrobial competition and predation. We identified 24 genes related to motility and gliding, including adventurous gliding motility proteins (e.g., GltC, GltG) and Cgl proteins (CglB, CglE, CglF), which likely underlie the social “wolf-pack” hunting behavior of *Myxococcus* and contribute to directed movement toward fungal prey.

### 3.3 Antagonistic and predatory activity against phytopathogenic fungi

**Figure 4:**
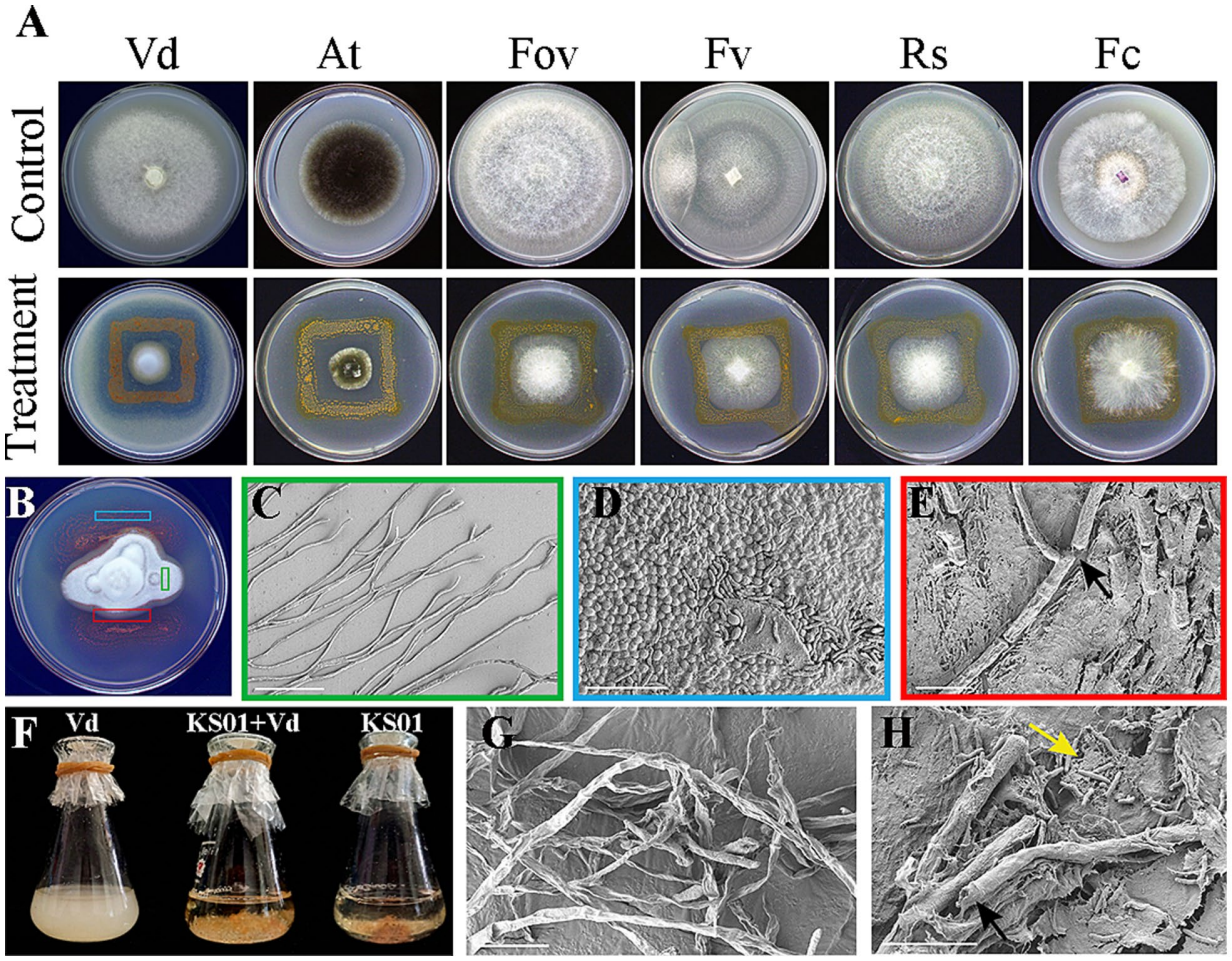
Antagonism and predation of strain KS01 against multiple phytopathogenic fungi. **(A)** Antagonistic activity of KS01 on VY/2 agar against plant pathogens: Vd, *Verticillium dahliae*; At, *Alternaria tenuissima*; Fov, *Fusarium oxysporum* f. sp. *vasinfectum*; Fv, *Fusarium verticillioides*; Rs, *Rhizoctonia solani*; Fc, *Fusarium culmorum*. **(B)** Predation of KS01 on *V. dahliae* on VY/2 agar. Green, blue and red boxes denote regions sampled for microscopic observation as C, D, and E, respectively. **(C)** Hyphae of *V. dahliae* not exposed to KS01 (control). Scale bar = 50 μm. **(D)** KS01 vegetative cells not engaged in predation, showing most of them differentiation to myxospores. Scale bar = 10 μm. **(E)** Hyphal fragmentation of *V. dahliae* following contact with KS01. Black arrows indicate sites of hyphal breakage. Scale bar = 10 μm. **(F)** Liquid cultures of *V. dahliae*, KS01, and their co-culture. **(G)** *V. dahliae* hyphae after 48 h in liquid culture (control). Scale bar = 10 μm. **(H)** Co-culture of KS01 and *V. dahliae* after 48 h showing hyphal fragmentation; yellow arrows indicate reticulate extracellular material secreted by KS01, black arrows indicate broken *V. dahliae* hyphae. Scale bar = 10 μm. Each treatment comprised three replicate plates, and the experiment was repeated three times.

In dual-culture assays on VY/2 agar, KS01 inhibited all six tested phytopathogens (*V*. *dahliae*, *A. tenuissima*, *F*. *oxysporum* f. sp. *vasinfectum*, *F. verticillioides*, *R. solani*, *F. culmorum*), with the strongest inhibition observed against *V. dahliae* (Figure 4A).

On solid medium KS01 exhibited active predation: KS01 cells spread toward and densely surrounded *V. dahliae* hyphae, causing hyphal collapse, extensive fragmentation and formation of reticulate extracellular material at contact zones (Figures 4B,E). Non-predating KS01 cells differentiated into spherical myxospores (Figure 4D), whereas control *V. dahliae* hyphae remained intact (Figure 4C). In liquid co-culture, *V. dahliae* growth and sporulation were markedly reduced and the culture supernatant became clear; extensive hyphal breakage was observed in treated samples but not in controls (Figures 4F–H).

### 3.4 Biocontrol efficacy and plant-growth promotion of KS01 solid formulation

**Figure 5:**
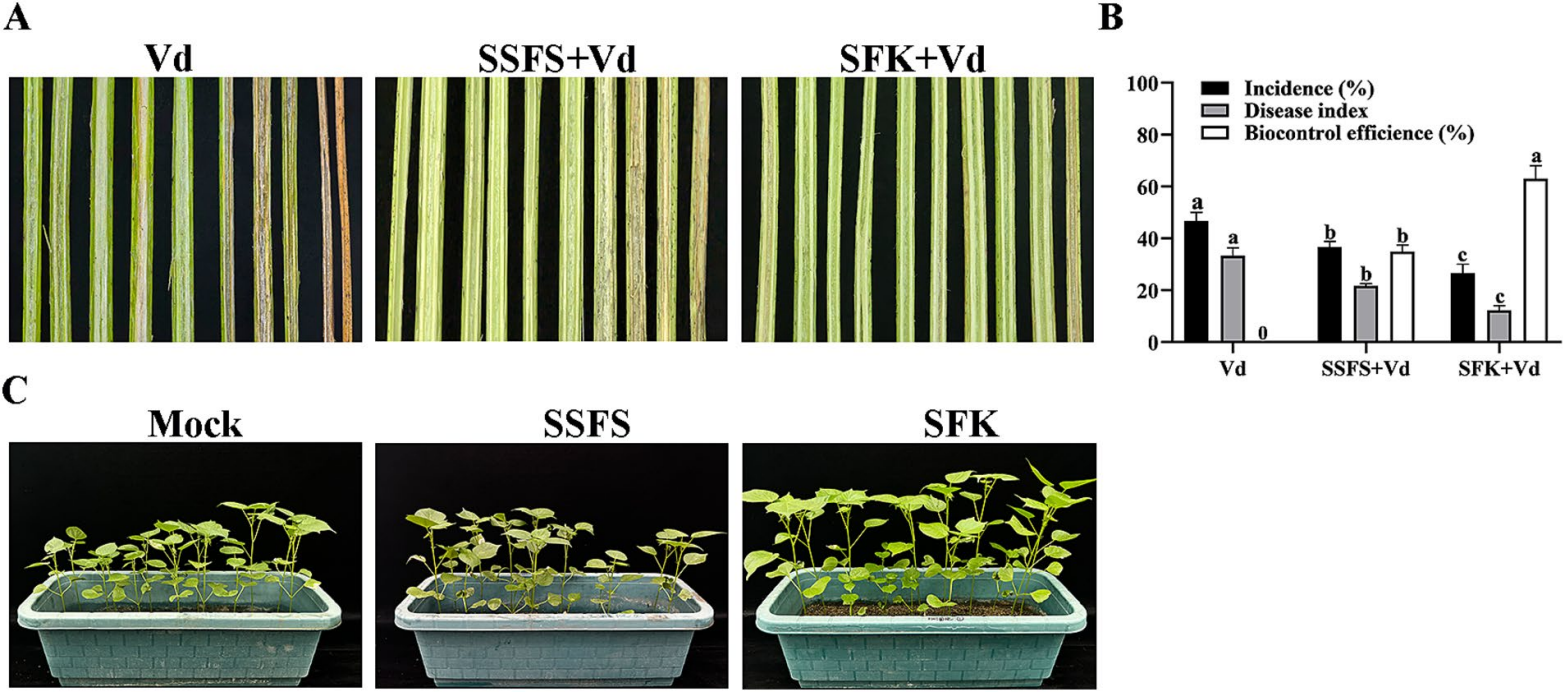
Biocontrol efficacy and plant growth promotion by strain KS01 against cotton Verticillium wilt. **(A)** Longitudinal sections of cotton stems under different treatments. Vd: inoculation with *V. dahliae* spore suspension (1.0 × 10^8^ spores·mL^−1^); SSFS: sterile solid fermentation substrate; SFK, solid fermentation product containing KS01. The disease lined from light to heavy from left to right in each panel. **(B)** Disease incidence, disease index and control efficacy (%) for each treatment (mean ± SD, *n* = 90). **(C)** Plant growth promotion effect of the KS01 solid formulation on cotton. Three pots for each treatment, 10 plants for each pot. The experiment was conducted in triplicate. Error bars represent standard deviations (±SD) of three replicates. Statistical significance was determined using Duncan’s multiple range test (*p* < 0.05). Bars sharing the same letter are not significantly different.

In greenhouse pot trials, treatment with the KS01 solid formulation (SFK) markedly reduced Verticillium wilt symptoms compared with the infected control (Vd). Vd plants showed severe vascular discoloration and plant death, whereas SFK-treated plants displayed only minor vascular discoloration (Figure 5A). Compared with the infected control (incidence 46.67%; disease index 33.33), SFK reduced disease incidence to 26.67% and disease index to 12.33% (*p* < 0.05), corresponding to a control efficacy of 63.01% (Figure 5B).

Under non-inoculated conditions, application of the KS01 solid formulation significantly increased plant height, stem diameter and primary root length relative to Mock and SSFS treatments (Figure 5C, Table 1), indicating a growth-promoting effect of KS01.

**Table 1:** Effects of KS01 solid fermentation production cotton growth parameters under greenhouse conditions.

Treatment	Plant height (cm)	Stem diameter (cm)	Primary root length (cm)	Shoot fresh weight (g)	Root fresh weight (g)	Shoot dry (g)	Root dry weight (g)
SFK	49.97 ± 3.30a	0.31 ± 0.02a	33.41 ± 1.62a	10.87 ± 0.91a	2.11 ± 0.14a	3.02 ± 0.37a	0.97 ± 0.11a
SSFS	40.32 ± 3.04b	0.26 ± 0.01b	29.50 ± 0.64b	7.26 ± 0.83b	1.79 ± 0.10ab	1.79 ± 0.14b	0.80 ± 0.04ab
Mock	35.19 ± 1.20b	0.25 ± 0.02b	25.72 ± 0.42c	5.43 ± 0.42b	1.58 ± 0.10b	1.57 ± 0.15b	0.61 ± 0.10b

SFK, solid fermentation product of strain KS01; SSFS, sterile solid fermentation substrate; Mock, untreated control. Each treatment consisted of 30 plants with three independent replicates. Different letters within a column indicate significant differences (*p* < 0.05) according to Duncan’s multiple range test.

### 3.5 Effects of cell-free fermentation filtrate and VOCs on *V. dahliae*

**Figure 6:**
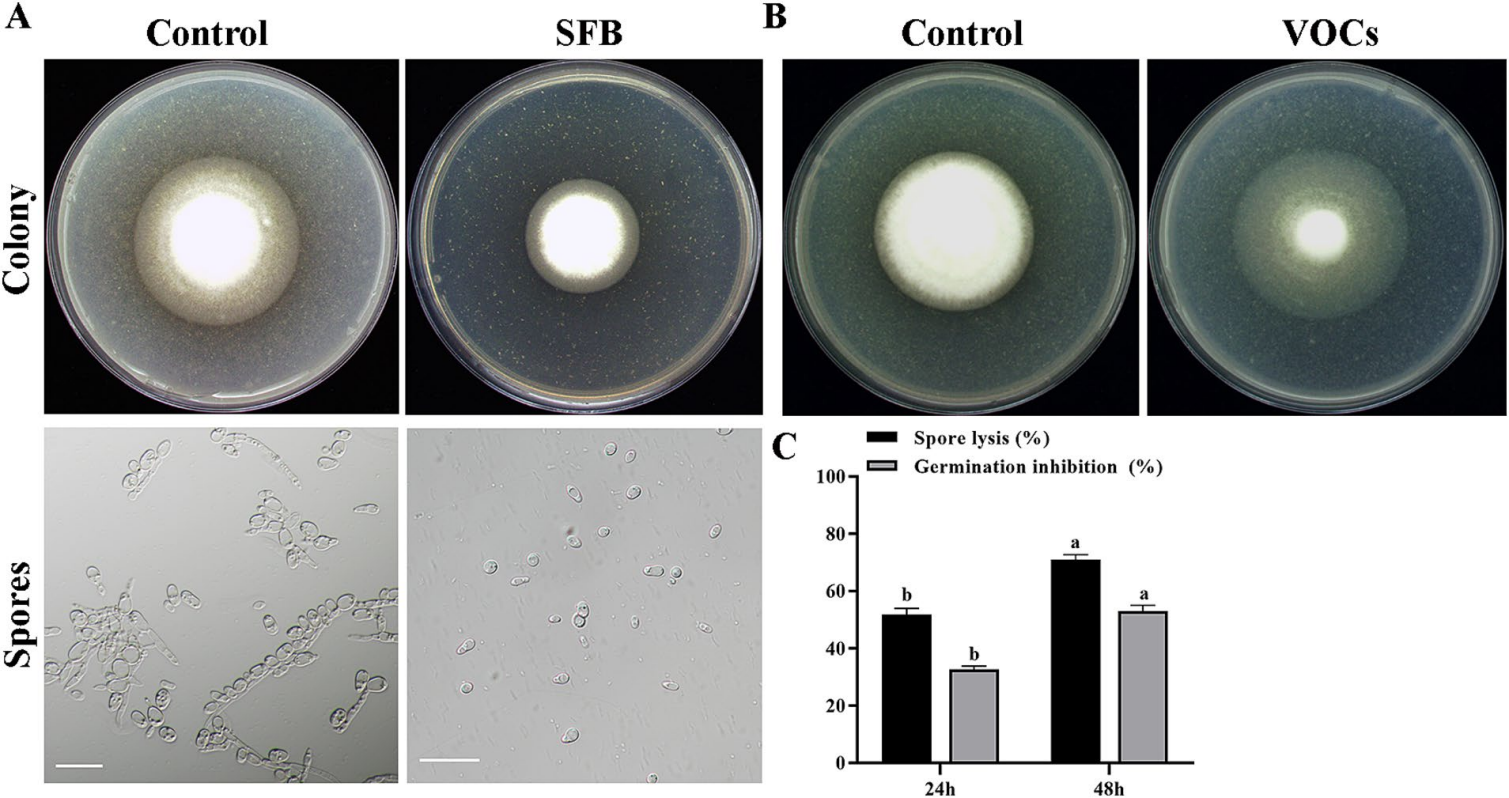
Inhibitory effects of KS01 sterile fermentation broth and volatile organic compounds (VOCs) on *Verticillium dahliae*. **(A)** Effects of sterile fermentation broth (SFB) on colony growth and spore germination of *V. dahliae* on VY/2 agar and under light microscopy. Scale bar = 10 μm. **(B)** Effects of KS01-derived VOCs on *V. dahliae* colony growth in a sealed plate assay. **(C)** Spore lysis rate and germination inhibition of *V. dahliae* treated with KS01 sterile fermentation broth at different exposure times. Error bars indicate standard deviation (±SD) from three independent replicates. Statistical comparisons were performed using Duncan’s multiple range test (*p* < 0.05); groups sharing the same letter are not significantly different.

Cell-free fermentation filtrate from KS01 significantly inhibited *V. dahliae* colony growth and caused spore lysis and germination inhibition (Figure 6A); quantitatively, spore lysis rates were 51.87 and 70.93% at 24 h and 48 h, respectively; corresponding spore germination inhibition rates were 32.65% (24 h) and 52.92% (48 h) (Figure 6C). Volatile organic compounds (VOCs) produced by KS01 also significantly suppressed hyphal growth in the sealed-plate assay (Figure 6B).

### 3.6 Antifungal activity of secreted fractions: metabolites, lipopeptides and crude protein

**Figure 7:**
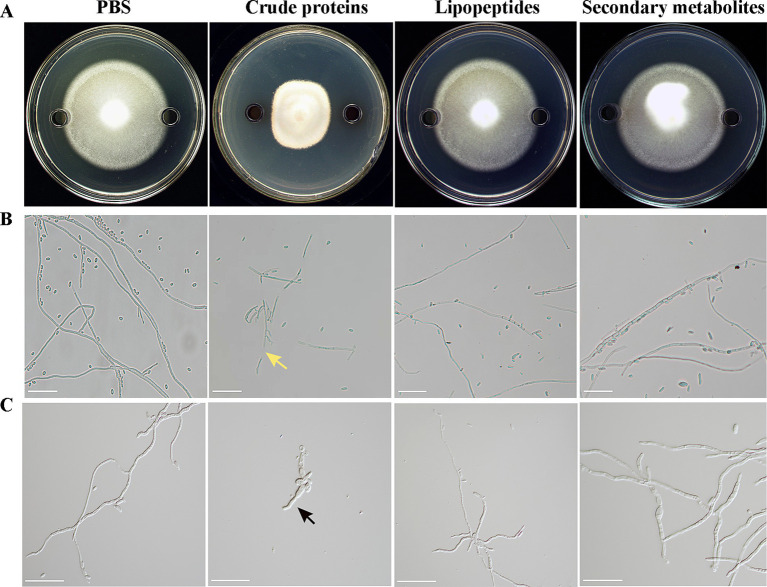
Antifungal activity of KS01 extracellular metabolites against *Verticillium dahliae*. **(A)** Co-culture of extracellular metabolites and *V. dahliae* on PDA. **(B)** Microscopic structural alterations in *V. dahliae* hyphae after treatment with extracellular metabolites; yellow arrows indicate hyphal fragmentation. Scale bar = 10 μm. **(C)** Microscopic observations of spore germination after treatment; black arrows indicate inhibition of spore budding. Scale bar = 10 μm. There were three biological replicates, each with three technique replicates.

Crude extracts of secreted secondary metabolites (100 mg·mL^−1^), lipopeptides (500 mg·mL^−1^) and extracellular proteins (3.1 mg·mL^−1^) were assayed by Oxford-cup and liquid exposure methods. Neither the crude lipopeptide nor the secondary-metabolite extract exhibited detectable inhibitory activity against *V. dahliae* under our assay conditions (Figures 7A–C). By contrast, the crude extracellular protein fraction produced pronounced antifungal effects: treated colonies showed hyphal collapse and extensive fragmentation at colony margins, and spore numbers and germ-tube growth were markedly reduced after protein treatment (Figures 7B,C).

### 3.7 Crude extracellular proteins compromise cell-wall and membrane integrity and induce ROS accumulation in *V. dahliae*

**Figure 8:**
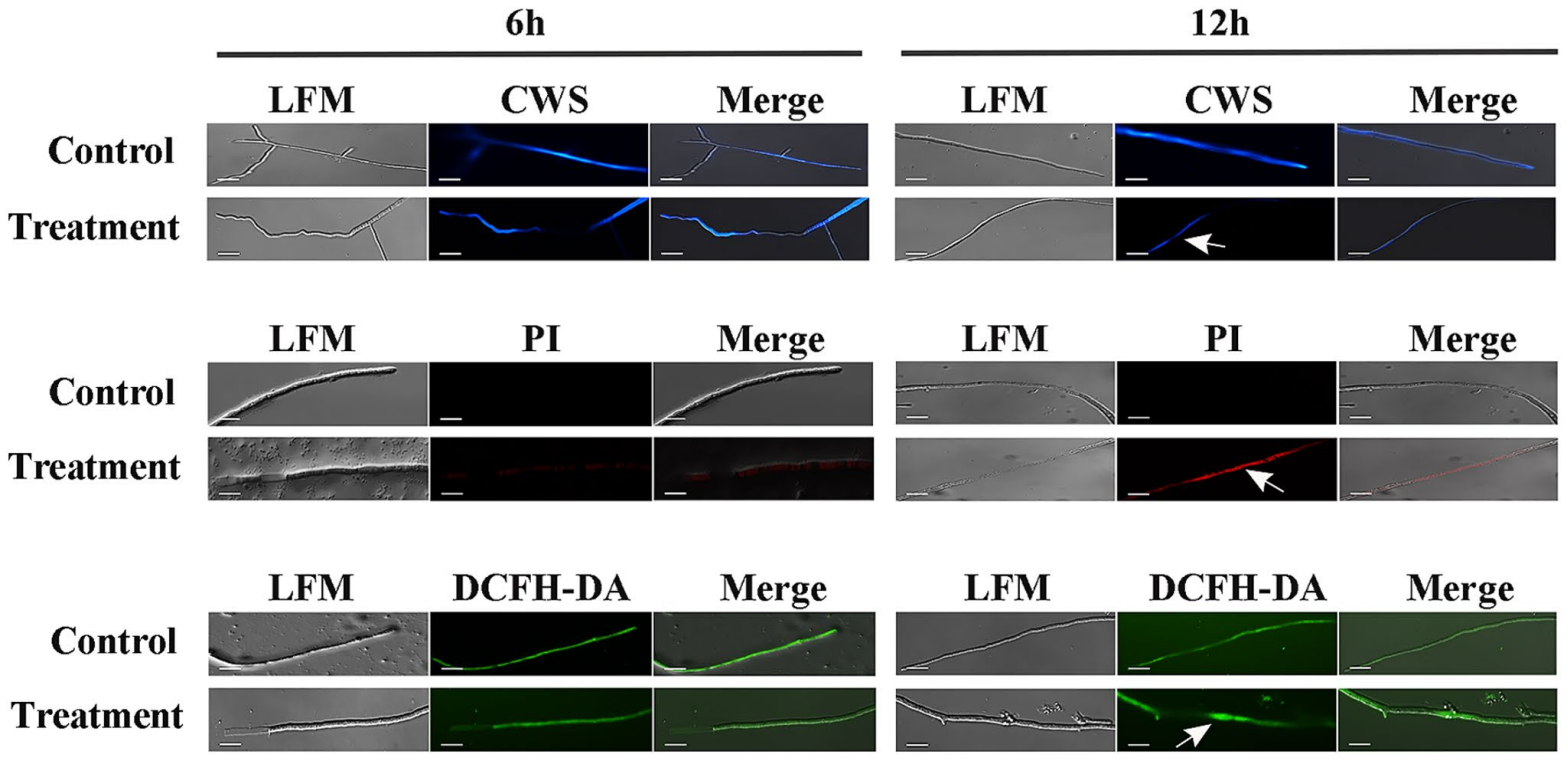
Effects of crude extracellular proteins from KS01 on cell-wall integrity, membrane integrity, and ROS accumulation in *Verticillium dahliae*. Cell-wall integrity was assessed by Calcofluor White staining; membrane integrity was assessed by propidium iodide (PI) staining; ROS accumulation was detected using the DCFH-DA fluorescent probe. Scale bar = 10 μm. LFM, bright-field microscopy; Merge, overlay of bright-field and fluorescence images. White arrows indicate hyphal discoloration or structural alteration. There were three biological replicates, each with three technique replicates.

Calcofluor White staining demonstrated progressive loss of cell-wall fluorescence in *V. dahliae* hyphae co-incubated with KS01 extracellular protein, indicating disruption of cell-wall integrity over time (Figure 8). Propidium iodide staining revealed accumulation of red fluorescence beginning at 6 h and increasing at 12 h, consistent with loss of membrane integrity and PI penetration. DCFH-DA staining showed significant intracellular reactive oxygen species (ROS) accumulation at 6 h, which intensified by 12 h. Together, these data indicate that extracellular proteins secreted by KS01 damage the fungal cell wall and membrane and trigger ROS bursts that likely contribute to cell death (Figure 8).

### 3.8 Extracellular enzyme activities and substrate spectrum of KS01

**Figure 9:**
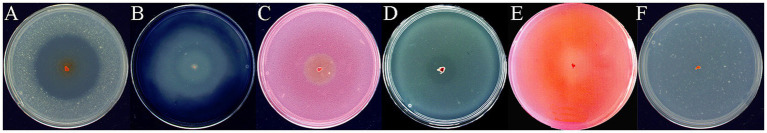
Hydrolytic enzyme activities of KS01 on different substrates. **(A)** Protease activity. **(B)** Amylase activity. **(C)** Cellulase activity. **(D)** Lipase activity. **(E)** Glucanase activity. **(F)** Chitinase activity. Assays were performed using substrate-specific plate assays. Zones of clearance or dye changes indicate enzymatic activity. There were three biological replicates, each with three technique replicates.

Qualitative plate assays showed KS01 can hydrolyze starch, skim milk, carboxymethyl cellulose, tributyrin and *β*-glucan (Figures 9A–E), while chitin remained resistant (Figure 9F). Quantitative DNS-based assays of the crude extracellular enzyme preparation demonstrated hydrolytic activity against xylan, β-1,3-glucan, yeast glucan, carboxymethyl cellulose and laminarin, but not against chitin or pustulan (Table 2). Activities were calculated against glucose and p-nitrophenol standard curves (glucose: *y* = 0.1393*x* + 0.0006, *R*^2^ = 0.9905; p-nitrophenol: *y* = 0.0398*x* − 0.0263, *R*^2^ = 0.993). The highest specific activities observed were xylanase activity (β-1,4-xylopyranosyl hydrolysis) at 53.37 ± 0.09 U·mL^−1^ and lipase activity (p-nitrophenyl palmitate hydrolysis) at 51.00 ± 0.14 U·mL^−1^ (Table 2). The cell wall of *V. dahliae* is primarily composed of β-1,3-glucans, chitin, and various hemicellulosic polysaccharides. The high xylanase activity exhibited by strain KS01 may facilitate the degradation of hemicellulose components within the fungal cell wall matrix, while glucanases target the β-glucan backbone. These synergistic enzymatic activities likely contribute to the disruption of cell wall integrity and subsequent hyphal lysis. In addition, the cell membrane of *V. dahliae*, which is rich in phospholipids and ergosterol, may be compromised by lipase-mediated degradation of membrane lipids, leading to the loss of membrane integrity observed in this study (Figure 8, PI staining).

**Table 2:** Hydrolytic activities of crude enzyme extract from strain KS01 against different substrates.

Substrates	Bond types	Enzyme activities (U·mL^−1^)
p-Nitrophenyl palmitate	Ester linkage	51.00 ± 0.14
Xylan	β-1,4-(xylopyranosyl)	53.37 ± 0.09
β-1,3-glucan	β-1,3-(glucose)	29.40 ± 0.02
Yeast glucan	β-1,3-β-1,6-(glucose)	26.45 ± 0.08
Carboxymethyl cellulose	β-1,4-(glucose)	2.44 ± 0.09
Laminarin	β-1,3-β-1,6-(glucose)	1.62 ± 0.13
Chitin	β-1,4-N-acetylaminoglycoside bond	0
Pustulan	β-1,6-(glucose)	0

## 4 Discussion

The widespread reliance on synthetic agrochemicals has intensified problems such as pathogen resistance, pesticide residues and soil degradation, motivating the search for environmentally benign alternatives. In this context, myxobacteria—social, predatory bacteria that produce diverse hydrolytic enzymes and secondary metabolites—represent an underexploited resource for crop protection (Contreras-Moreno et al., 2024; Zhang et al., 2024). This study isolated a myxobacterial strain, KS01, from saline–alkaline cotton soil and identified it as *M. fulvus* based on morphology and multilocus sequence analysis. KS01 displayed broad-spectrum antagonism *in vitro* and provided substantial control (63.01%) of cotton Verticillium wilt in greenhouse trials, indicating clear potential as a biocontrol agent.

Early work attributed the predatory capacity of myxobacteria primarily to the secretion of hydrolytic enzymes and secondary metabolites (Berleman and Kirby, 2009). More recently, attention has expanded to the roles of diverse enzyme classes. Genomic analyses have revealed that myxobacterial genomes encode abundant peptidases, glycoside hydrolases, polysaccharide lyases, and carbohydrate esterases (Whitworth et al., 2021; Saraf and Sharma, 2025), which are likely to contribute significantly to predation. For example, *β*-1,6-glucanase, *β*-1,3-glucanase and chitinase secreted by *Corallococcus* sp. EGB can hydrolyze glycosidic bonds in the cell walls of plant pathogens, thereby compromising cell-wall integrity (Li et al., 2019; Zhou et al., 2019). Given the broad prey spectrum of myxobacteria and their ability to completely remove prey biomass, they likely possess a multifunctional predatory toolkit that enables nutrient acquisition from taxonomically diverse prey and targets multiple structural components of prey cells (Zhou et al., 2023). Our findings are consistent with these previously reported, multifaceted mechanisms. The strain KS01’s biocontrol mechanisms appear to be multifactorial. We observed active predation on *V. dahliae*—KS01 cells aggregated at hyphal surfaces, produced reticulate extracellular material at contact zones, and induced extensive hyphal fragmentation in both solid and liquid assays (Figure 4). In addition to contact-dependent effects, KS01 produced volatile organic compounds (VOCs) and cell-free fermentation filtrates that inhibited colony growth and reduced spore viability and germination (Figure 6). Importantly, biochemical fractionation showed that the crude extracellular protein fraction, rather than crude lipopeptides or the broad secondary-metabolite extract, was the principal fungicidal component under our assay conditions. Fluorescent staining revealed that this protein fraction compromises fungal cell-wall and membrane integrity and triggers intracellular ROS accumulation—events consistent with enzyme-mediated lysis and oxidative damage (Sharma et al., 2025).

KS01 secretes a rich complement of hydrolytic activities (glucanase, cellulase, amylase, lipase, and protease) and exhibits strong xylanase and lipase activities in crude extracts. Such enzyme suites are congruent with a predatory lifestyle that requires degradation of diverse macromolecular substrates in prey cell walls and membranes and have been implicated in myxobacterial predation in other studies (Li et al., 2019; Zhou et al., 2019). While many studies emphasize secondary metabolites in myxobacterial antagonism, our results underscore the central role that extracellular proteins and hydrolytic enzymes can play in fungal lysis and disease suppression.

Although studies on the application of myxobacteria in the biological control of plant diseases remain limited, accumulating evidence suggests their considerable potential. For example, Ye et al. (2020b) reported that a solid formulation based on *Corallococcus* sp. EGB achieved a stable biocontrol efficacy of approximately 50% against cucumber Fusarium wilt over two consecutive years of field trials. Compared with frequently used biocontrol taxa (e.g., *Bacillus*, *Streptomyces*), KS01’s active predation may confer advantages in complex soils where direct removal of pathogen biomass and local restructuring of the microbial community are desirable. By comparison with conventional biocontrol bacteria used against cotton Verticillium wilt, *Bacillus amyloliquefaciens* E2 achieved approximately 66.67% control efficacy under pot conditions (Du et al., 2026). *Bacillus velezensis* BvZ45-1 showed >46% efficacy under both greenhouse and field conditions (Sun et al., 2024). *Streptomyces rectiviolaceus* 2–59 reached a pot-trial control efficacy of 62.72% and exhibited plant growth-promoting effects (Zhang et al., 2026). Unlike these traditional biocontrol strains, the active predatory mechanism of *M. fulvus* KS01 differs fundamentally from antagonism and may confer a competitive advantage in complex soil environments (Munoz-Dorado et al., 2016). The observed greenhouse efficacy of KS01 is within the range reported for those conventional biocontrol strains. However, there remains substantial room for improvement. Several factors may have influenced the observed biocontrol efficacy. First, the solid-state fermentation process using larval frass of *P. brevitarsis* as the substrate has not yet been systematically optimized. Key parameters, including moisture content, inoculum size, carbon-to-nitrogen ratio, and fermentation duration, may affect both the viable cell density and metabolic activity of the biocontrol agent. Second, the application rate and timing likely require further optimization to ensure adequate colonization of strain KS01 at infection sites, particularly during the critical stages of *V. dahliae* invasion of cotton roots and subsequent colonization of the vascular tissues. Nevertheless, its performance must be validated across diverse soils, cotton cultivars and multi-season field trials to assess robustness and agronomic utility.

Furthermore, KS01 also promoted plant growth in the absence of pathogen challenge. The ability of strain KS01 to produce indole-3-acetic acid (IAA), solubilize phosphate, and fix nitrogen was evaluated using the Salkowski assay (Guardado-Fierros et al., 2024), Pikovskaya agar plate method (Bansal et al., 2025), and growth on Ashby’s nitrogen-free medium (Liu et al., 2017), respectively. The results indicated that strain KS01 did not exhibit any of these activities (data not shown), we propose several plausible indirect mechanisms exist based on our research and literatures (Zhang et al., 2012; Wang W. et al., 2020; Chen et al., 2023; Dai et al., 2024): (i) secretion of extracellular enzymes could accelerate organic matter turnover and increase local availability of nutrients for the plant and beneficial microbes; (ii) predation could suppress deleterious microbes and thereby improve rhizosphere quality; and (iii) interactions with other soil microbes might enhance nutrient cycling (e.g., nitrogen mineralization). Disentangling these indirect effects will require targeted rhizosphere microbiome profiling, metabolomics and nutrient-cycling assays.

There are several limitations to the present study and clear priorities for follow-up work. First, although crude extracellular protein is the dominant antifungal fraction in our assays, the active molecule(s) remain unidentified—proteomic fractionation, targeted enzymology, heterologous expression and loss-of-function studies (gene knockout or CRISPRi) are needed to pinpoint causal effectors. Second, VOCs and small-molecule BGC products detected by antiSMASH should be chemically characterized (GC–MS, LC–MS/MS) to define their contribution under field-relevant conditions. Third, the ecological consequences of introducing a predatory bacterium into agricultural soils (non-target effects, persistence, horizontal gene transfer) must be assessed via controlled field trials and biosafety evaluations. Finally, formulation optimization (carrier selection, shelf life, co-inoculants) and dose–response studies are required to translate greenhouse efficacy to consistent field performance.

In conclusion, *M. fulvus* KS01 exhibits a combination of contact-mediated predation, enzyme-driven lysis and diffusible antagonism that together suppress *V. dahliae* and promote cotton growth under greenhouse conditions. These properties make KS01 a promising candidate for development as a biological control agent against Verticillium wilt. Future work should prioritize molecular identification of active effectors, comprehensive field evaluation across environments and formulation development to enable practical agricultural deployment.

## 5 Conclusion

In this study, a predatory myxobacterial strain, KS01, was successfully isolated and identified as *M. fulvus* based on morphological characteristics and molecular phylogenetic analyses. The strain exhibited strong antagonistic activity against *V. dahliae in vitro*. Greenhouse pot experiments further demonstrated that a solid formulation of *M. fulvus* KS01, prepared using white star flower chafer (*P. brevitarsis*) frass as the fermentation substrate, provided effective control of cotton Verticillium wilt and significantly promoted plant growth, highlighting its practical application potential. Mechanistic investigations indicate that extracellular enzymes secreted by KS01—particularly lipases, peptidases, and glycoside hydrolases—likely play pivotal roles in fungal predation and pathogen suppression. Overall, this work introduces *M. fulvus* KS01 as a promising predatory biocontrol resource and provides a novel strategy for the sustainable management of cotton Verticillium wilt.

## Data Availability

The datasets for this study can be found in the NCBI database with BioProject: PRJNA1424368, BioSample: SAMN55382687.
