# Supplementary material for: Predatory Myxococcus fulvus strain KS01 confers biocontrol and growth promotion in cotton through multifaceted antifungal mechanisms
Source: Front Microbiol. 2026 Jun 17;17:1812103. doi: 10.3389/fmicb.2026.1812103 (PMC13318889; doi:10.3389/fmicb.2026.1812103)
Supplement: Supplementary file 1 [file Table_1.docx]

Supplementary Material

# Supplementary Figure 1


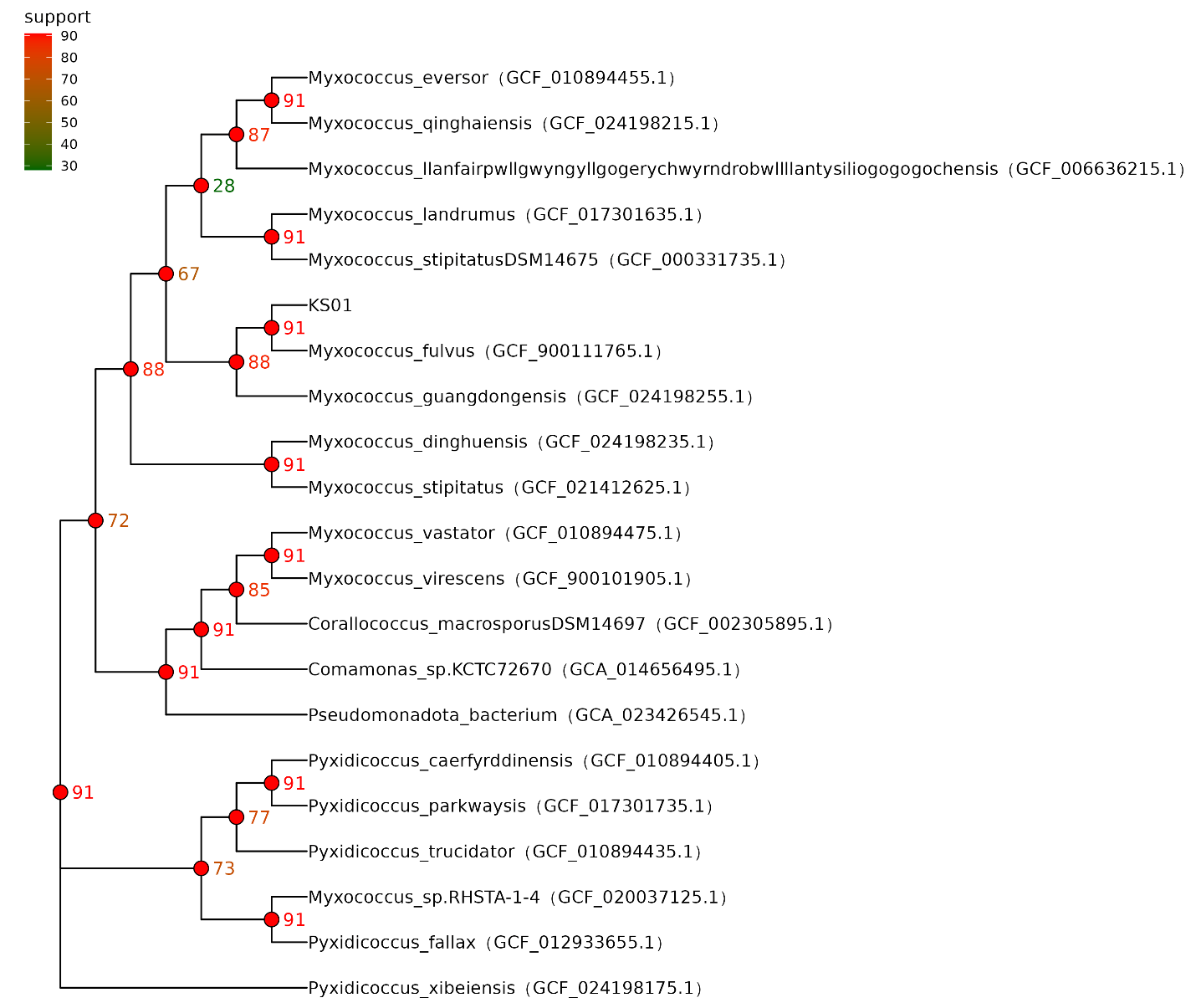


**Supplementary Figure 1.** Phylogeny based on 92 core genes. Node labels indicate the number of genes supporting each branch.
